# Successful Preservation of Native BCR::ABL1 in Chronic Myeloid Leukemia Primary Leukocytes Reveals a Reduced Kinase Activity

**DOI:** 10.3389/fonc.2022.904510

**Published:** 2022-06-08

**Authors:** Christian Boni, Massimiliano Bonifacio, Marzia Vezzalini, Luigi Scaffidi, Luisa Tomasello, Laurie L. Parker, Diego Boscarino, Dino Paladin, Mauro Krampera, Claudio Sorio

**Affiliations:** ^1^ Department of Medicine, General Pathology Section, University of Verona, Verona, Italy; ^2^ Department of Medicine, Hematology Section, University of Verona, Verona, Italy; ^3^ Department of Biochemistry, Molecular Biology and Biophysics, Masonic Cancer Center, University of Minnesota, Minneapolis, United States; ^4^ AB Analitica, Padova, Italy

**Keywords:** chronic myelogenous leukemia, acute lymphocytic leukemia, philadelphia chromosome, Bcr Abl, tyrosine kinase, imatinib (Gleevec), kinase activity, kinase assay

## Abstract

Chronic myeloid leukemia (CML) is a myeloproliferative disease caused by the acquisition of t(9;22) generating the fusion tyrosine kinase BCR::ABL1. However, despite the crucial role of this protein in the dysregulation of numerous signal transduction pathways, a direct measure of BCR::ABL1 kinase activity in chronic phase (CP) CML was never accomplished due to intense degradative activity present in mature leukocytes. Therefore, we developed a procedure suitable to preserve BCR::ABL1 protein under non-denaturing, neutral pH conditions in primary, chronic phase (CP)-CML samples. As a result, specific kinase activity was detected utilizing a biotinylated peptide substrate highly selective for c-ABL1. Furthermore, through this approach, BCR::ABL1 kinase activity was barely detectable in CP-CML compared to Ph^+^ acute lymphoblastic leukemia primary samples, where kinase activity is comparable to those measured in Ph^+^ cell lines. These *in vitro* findings provide the first direct measure of BCR::ABL1 kinase activity in primary CP-CML and reveal the presence of a still uncharacterized inhibitory mechanism that maintains BCR::ABL1 in a low activity state in CP-CML despite its overexpression.

## Introduction

Reportedly from literature, at least one third of patients who initially respond to tyrosine kinase inhibitor (TKI) treatment relapsed due to the BCR::ABL1 kinase activity independence of primitive chronic myeloid leukemia cells (1–3). Characterizing this phenomenon has been challenging—while intact BCR::ABL1 can be easily isolated from cultured Ph^+^ cell lines (*e*.g., K562), its recovery from primary cells in non-denaturing lysis conditions typically fails due to the presence of intense degradative activity in mature PMNs, requiring denaturing lysis conditions to preserve the protein (4, 5). However, denaturation precludes the direct analysis of enzymatic activity (6, 7). Consequently, despite decades of research on BCR::ABL1 protein, its enzymatic activity in CP-CML has never been directly measured in primary leukocytes. This widely recognized issue has led to the development of alternative/indirect assays that measure BCR::ABL1 activity by detecting a downstream-phosphorylated substrate such as p-CRKL (8–12) with no direct demonstration that its phosphorylation is due to BCR::ABL1 itself or to other activated tyrosine kinase/s present and active in the cells.

Measuring BCR::ABL1 activity (as opposed to just the protein levels) in primary cells could be a valuable way to test the kinase inhibitor efficacy at the beginning of the patient’s treatment (13), but methods to directly assay it are still a need in the field. A specific cell-based assay suitable for measuring endogenous BCR::ABL1 kinase activity has been described but awaits validation in primary leukemia samples (14–16). Therefore, we sought to devise a non-denaturing cell lysis method capable of protecting the critical targets in CML primary leukocytes, predominantly in the protease-rich fraction represented by myeloid cells, from proteolysis (in this specific case including, but not restricted to, BCR::ABL1), followed by a specific kinase assay. Following setup and validation in the CML cell lines, we successfully applied these procedures on samples derived from primary Ph^+^ leukemia samples [CML and acute lymphoblastic leukemia (ALL)].

## Methods

### Cell Lines

Murine pro-B cell line Ba/F3 cells were maintained in RPMI 1640 (Life Technologies, Inc., Gaithersburg) medium supplemented with 10% heat-inactivated fetal bovine serum (FBS), 2 mM L-glutamine, and 10% conditioned medium from WEHI cell culture producing murine recombinant IL-3 and incubated at 37°C with 5% CO_2_ atmosphere. K562 cell lines were cultured as detailed above without addition of murine IL-3. All cell lines were routinely tested for mycoplasma contamination.

### Kinase Inhibitors

Imatinib (STI-571) and dasatinib (BMS-354825) were purchased from Selleckchem (Houston, TX, USA). Stock solutions of imatinib and dasatinib at 1 or 10 mM in sterile water were filtered and stored at −20°C. For imatinib (1 µM) and dasatinib (1 nM) exposure, the indicated concentration was added to the cell cultures for the indicated times before harvesting the cells for Western blot analysis and immunoprecipitation experiments.

### Patients’ Population

The patients in this study were enrolled onto the trial “Studio sperimentale esplorativo sul monitoraggio PRecoce mediante biosensore dell’attività degli INibitori delle Tirosin chinasi in pazienti con leucemia mieloide cronica (SPRINT, protocol 1053CESC)”—a study conducted on adult patients with newly diagnosed CML. EDTA-anticoagulated peripheral blood (PB) cells were obtained from newly diagnosed patients with CP CML. All patients were enrolled within 7 days of diagnosis, without history of TKIs or interferon therapy.

### Thawing Procedure for the Patient Samples

ALL Ph^+^ samples were kindly provided by Prof. Bonifacio (Hematology Section of the Department of Medicine, Verona). Briefly, after the standard Ficoll separation of the leukocyte fraction from the bone marrow aspirate, cells were collected by the interface, diluted in RPMI 1640, centrifuged at 200 × *g* for 10 min and resuspended in RPMI1640, 30% v/v FBS and 10% v/v DMSO. The samples were transferred in cryovials, stored overnight at -70°C in a Cryobox™, and then transferred in liquid nitrogen vapor phase. Samples were thawed in Iscove Modified Dulbecco Media at 37°C containing 10 U/ml of deoxyribonuclease I (Sigma, St. Louis, MI, USA) to avoid clumping. The cells were centrifuged at 200 × *g* for 15 minutes and then treated according to the LeukoProtect protocol, as described below. The featured patients are summarized in **Tables 1** and **2**.

**Table 1:** Characteristics of the chronic myeloid leukemia patients.

	Age	M/F	Hb (g/dl)	WBC (10^9^/L)	Cytogenetics	BCR::ABL1	SOKAL risk
**CML #1**	40	M	14.8	42.35	46,XY,t(2;9:22)(p15;q11)	b3a2	Low
**CML #2**	47	F	12.2	16.21	46,XX,t(9;22)(q34;q11)	b3a2	Low
**CML #3**	66	M	15.8	33.07	46,XX,t(9;22)(q34;q11)	b2a2	Low
**CML #4**	75	M	12.7	23.13	46,XX,t(9;22)(q34;q11)	b2a2	Intermediate
**CML #5**	65	M	10.2	28.36	46,XX,t(9;22)(q34;q11)	b2a2	Low
**CML #6**	73	F	11.4	19.88	46,XX,t(9;22)(q34;q11)	b3a2	Intermediate
**CML #7**	51	F	12.5	32.61	46,XX,t(9;22)(q34;q11)	b3a2	Intermediate
**CML #8**	72	M	14.8	27.87	46,XX,t(9;22)(q34;q11)	b3a2	Low
**CML #9**	82	F	13.3	27.68	46,XX,t(9;22)(q34;q11)	b3a2	Intermediate
**CML #10**	69	F	14.3	46.55	46,XX,t(9;22)(q34;q11)	b3a2	Intermediate
**CML #11**	50	F	12.8	65.03	46,XX,t(9;22)(q34;q11)	b2a2	Low

**Table 2:** Characteristics of the acute lymphoblastic leukemia patients.

	Age	M/F	Hb (g/dl)	WBC (10^9^/L)	Cytogenetics	PB blasts (%)	BM blasts (%)	BCR::ABL1
**ALL #1**	64	F	11.8	112.14	48,XX,+X,+6,t(9;22)(q34;q11)[11]/46,XX,t(9;22)(q34;q11)[9]	67%	48%	p190 (e1a2)
**ALL #2**	39	F	7.5	87.83	46,XX,t(9;22)(q34;q11)	57%	72%	p210 (b3a2)
**ALL #3**	34	F	10.6	43.6	46,XX,t(9;22)(q34;q11)[11]/46,XX[11]	79%	90%	p190 (e1a2)

### Cell Lysis and LEUKOPROTECT Protocol

Whole blood was suspended in Roche + LeukoProtect (R+L) pre-treatment buffer which was prepared as follows: Roche tablet, cOmplete™ ULTRA Tablets, Mini, EDTA-free (Sigma, St. Louis, MI), was resuspended in phosphate-buffered saline (PBS) as suggested by the manufacturer, and then LeukoProtect (AB Analitica, Padova, Italy) and 10 mM EDTA were added. After 20 min of incubation at room temperature (with continuous slow shaking), osmotic lysis of red blood cells (RBC) was performed (RBC lysis buffer: 0.155 M NH_4_Cl, 0.01 M KHCO_3_, and 0.1 mM EDTA in ddH_2_O). The cells were centrifuged at 200 × *g* for 5 min, the supernatant was removed, and the pelleted leukocytes (myeloid + lymphoid cell fraction) were lysed in an appropriate volume of cold lysis buffer (50 mM Tris-HCl, 150 mM NaCl, 1% Triton-X 100, 2 mM EDTA, and cOmplete™ ULTRA Tablets 1× in ddH_2_O). The samples were kept on ice for 15 min and then centrifuged at 13,000 × *g* at 4°C for 15 min. The protein concentration of the resulting cleared lysate was quantified using the Bradford protein assay (SERVA Electrophoresis, Heidelberg, Germany). The lysate aliquots were stored at -80°C.

### BCR::ABL1 Immunoprecipitation

Protein lysates were incubated with an antibody against total c-Abl (Cell-Signaling Technology, Danvers, MA, USA) for 2 h at 4°C on a rotating wheel. Protein G-Dynabeads (Invitrogen, Carlsbad, CA, USA) were added, and the mixture was incubated for 1 h at 4°C on wheel. The kinase assay (described below) was performed, and the beads containing BCR::ABL1 protein were then heated in Laemmli buffer for 5 min at 95°C to denature and elute the bound protein before separation on SDS–polyacrylamide gels.

### Kinase Assay

We utilized a peptide designed and validated as specific for ABL kinase (Sequence: EAIYAAPFAKK-biotinK-GGCGGAPTYSPPPPPG) (14, 17). After BCR::ABL1 immunoprecipitation, but before denaturation in Laemmli sample buffer, 1 µM substrate peptide (GenScript Biotech, Piscataway, NJ, USA) was resuspended in kinase assay buffer (20 mM Tris-HCl, 10 mM MgCl_2_, 0.05% Triton-X, 20 µM ATP, 1 mM DTT, and 0.1 mM Na_3_VO_4_ in H_2_O) and added to the immunocomplexes for 30 min on a rotating wheel at 4°C. The peptide was recovered from the supernatant *via* its biotinylated site by capture on NeutrAvidin™ Coated High-Capacity Plates (Thermo Fisher Scientific, Waltham, MA, USA). After three washes, mouse monoclonal anti-phosphotyrosine antibody, clone 4G10 (Millipore Corporation, Bedford, MA, USA), was added and then incubated at 4°C overnight. The plates were washed and incubated with anti-mouse IgG antibody conjugated with HRP for 1 h at room temperature. Amplex Ultra Red Reagent (Invitrogen, Carlsbad, CA, USA) developing solution was then added. The plates were analyzed in a Victor plate reader (PerkinElmer, Waltham, MA, USA) at 540-nm excitation and 572-nm emission wavelengths.

### Western Blotting

Protein samples in SDS–PAGE gels were transferred onto polyvinylidene fluoride membranes (Bio-Rad, Hercules CA). Blocking was performed using a solution of TBS 0.1% Tween 20/BSA 3%. Incubations with primary antibodies were done overnight at 4°C in a shaking system. Incubation with secondary horseradish peroxidase–conjugated anti-mouse IgG or anti-rabbit IgG antibodies (Cell-Signalling Technology, Danvers, MA, USA) was performed for 1 h. All antibodies were employed according to the manufacturer’s recommendations (details are shown in **Table 3**). Antibodies were detected using enhanced chemiluminescence with ECL Supernova (Cyagen, Santa Clara, CA, USA). All images were acquired with the ImageQuant^®^LAS 4000 biomolecular imager (GE Healthcare Europe GmbH, Germany). The bands were quantified to obtain optical density values as described below.

**Table 3:** List of antibodies.

Antibody	Code	Host	Company
**Phospho-c-Abl (Tyr245)**	#2861	Rabbit	Cell Signaling
**c-Abl**	#2862	Rabbit	Cell Signaling
**Jak2 (D2E12)**	#3230	Rabbit	Cell Signaling
**Syk (D3Z1E)**	#13198	Rabbit	Cell Signaling
**Btk**	Ab54129	Mouse	Abcam
**Akt**	#9272	Rabbit	Cell Signaling
**Lyn**	Sc-15 (44)	Rabbit	Santa Cruz
**Sapk/Jnk**	#9252	Rabbit	Cell Signaling
**Erk 1/2**	#9102S	Rabbit	Cell Signaling
**Mapk p38**	#9212	Rabbit	Cell Signaling
**Anti-phospho** **4G10 clone**	05-321	Mouse	Merk-Millipore
**β-Actin (13E5)**	#4970	Rabbit	Cell Signaling

### Optical Density Detection

The densitometry values of blotted bands were quantified using the ImageJ software and then normalized with the indicated housekeeping proteins. The optical density values for BCR::ABL1 immunoprecipitated from primary samples were standardized in ImageJ software using a calibration curve derived from different amounts of BCR::ABL1 immunoprecipitated from K562 cells in order to calculate the corrected values (**Figure S1**).

### Statistical Analysis

Analyses were performed using GraphPad 7 Instat software, and data are displayed as the mean ± SD. *P*-values were calculated using Student’s *t*-test for evaluation of differences between groups. Thresholds defined for statistically significant *p*-values are noted in each figure where relevant.

## Results

### BCR::ABL1 Activity Detection in Ph^+^ Cell Lines

**Figure 1:**
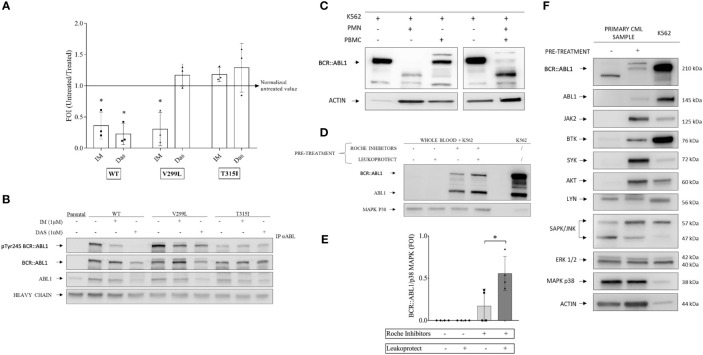
Kinase assay optimization and setup of a protease protection protocol. **(A)** BCR-ABL1 and variants transfected into mouse-derived pro-B Ba/F3 cell lines were treated with 1 μM imatinib and 1 nM dasatinib for 12 h alongside an aliquot of each cell type and non-transfected Ba/F3 maintained without tyrosine kinase inhibitors as controls. Scatter plots represent three independent experiments in which the kinase activity measurements were normalized to a total BCR-ABL1 protein amount detected by Western blot analysis of immunoprecipitated samples. One-way ANOVA multiple comparison was applied for all cell lines with a *p*-value significance threshold of < 0.05 applied. **(B)** Examples of Western blot analysis of immunoprecipitated BCL-ABL1 and variants from the experiments shown in **(A)**. **(C)** Polymorphonuclear neutrophils (PMNs) and peripheral blood mononuclear cells were collected from healthy donor blood. Each cell population was spiked with BCR-ABL1-positive cell line (K562) at 10:1 ratio. The cell mixture was lysed using a non-denaturing lysis buffer, including protease inhibitors, as described in the **Supplementary Information**. Total protein was quantified *via* the Bradford assay, and Western blot analysis was performed, showing that the PMN fraction contained a degradative activity. **(D)** Healthy whole-blood samples combined with K562 (ratio 10:1) were pretreated with Roche cOmplete™ ULTRA Tablets inhibitors alone, LeukoProtect alone, or the combination of both. K562 cell lysate was used as a reference. **(E)** Densitometry analysis of BCR-ABL1 protein detected by Western blot as shown in **(D)**. One-way ANOVA multiple comparison was applied, with a *p*-value significance threshold of < 0.05 (*) applied. **(F)** Combined Roche and LeukoProtect (R+L) pre-treatment protocol preserves several kinases from degradation. Western blot analysis of lysates from CP-CML pre-treated (+) or not **(-)** with R+L before lysis. The membrane was probed with the specific antibodies indicated. K562 cell lysate is loaded as a reference. *p< 0.05.

The conditions for the kinase assay protocol were developed initially in Ba/F3 cells stably expressing native and drug-resistant mutants of BCR::ABL1. Normalized kinase activity was consistent with the sensitivity of the mutant to TKIs (18) (**Figure 1A**). A Western blot of protein eluted from immunoprecipitation demonstrated that BCR::ABL1 was present and intact (**Figure 1B**). These results show that this assay can identify TKI sensitivity and resistance, supporting its efficacy for identifying drug-responsive and drug-resistant cells.

Our goal was to establish a protocol and the conditions that could overcome the known proteolytic degradation issue arising in PMN cell preparations (4–6). We first confirmed published reports that standard protease inhibitors used alone in non-denaturing cell lysis buffer were insufficient to inhibit the strong protease activity present in primary leukocytes (**Figure 1C**) (see the **Supplementary Methods** for a complete description).

We then used a mixture of healthy donor blood and K562 cells (10:1 ratio) as a model system to simulate primary CML samples and worked to develop conditions that could prevent degradation. Optimal protection was achieved by pre-treating the blood/K562 with a mixture of cOmplete™ ULTRA protease inhibitors (Roche) with LeukoProtect (AB Analitica) and EDTA in PBS (**Figures 1D, E**). These R+L pre-treatment conditions were then demonstrated with primary CML samples, successfully preserving the BCR::ABL1 protein from degradation (**Figure 1F**). Of note is that we also showed that other relevant kinases (*e*.*g*., AKT, BTK, JAK2, and SYK) are subject to degradation and protected by the pre-treatment conditions that we developed. In contrast, the other proteins (LYN, ERK1/2, p38 MAPK, and ACTIN) appear unaffected (**Figure 1F**).

### BCR::ABL1 Kinase Activity Measurement in CP-CML and ALL Ph^+^ Patient Samples

**Figure 2:**
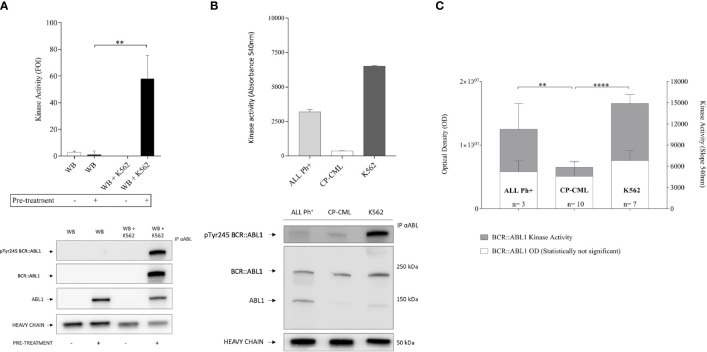
BCR-ABL1 kinase activity measurement in CP-CML and ALL Ph+ patient samples. **(A)** Immunoprecipitates obtained from healthy donor blood and the same blood samples mixed with K562 to simulate CML were processed with (+) or without **(-)** R+L pretreatment protocol and tested for specific kinase activity using the Abl substrate peptide. Kinase activity graph (top) and representative Western blot analysis (bottom) underline the preservation of both BCR-ABL1 protein integrity and function only after the R+L pre-treatment procedure. **(B)** Kinase activity assay (top) with BCR-ABL1 protein immunoprecipitated from 500 µg of CP-CML or ALL Ph^+^ primary leukocyte lysate, with K562 cell line as reference. Reduced kinase activity seen in the CP-CML sample compared to a cell line in BC-CML phase (K562) and to the ALL Ph^+^ sample (duplicates). Western blot analysis performed on total and P-Y245 BCR-ABL1 derived from the same immunoprecipitated samples whose activity was measured (bottom). **(C)** BCR-ABL1 kinase activity from 13 patient samples (including 10 CP-CML and 3 ALL Ph^+^) alongside K562 (*n* = 7 biological replicates). The values obtained were normalized by an immunoprecipitated protein amount measured by densitometry analysis (ImageJ software on western blot membrane probed with anti-ABL1-antibody). One-way ANOVA multiple comparison was applied with a *p*-value significance threshold of < 0.05 applied. **p = 0.0017; ****p < 0.0001.

We then used the protective R+L pre-treatment described above to prepare immunoprecipitated kinase from whole blood mixed with K562 cells at 10/1 ratio (or whole blood alone as a negative control) to simulate primary CML and performed the kinase assay. **Figure 2A** shows that the BCR::ABL1 activity is preserved only when the R+L pre-treatment is performed. The protocol (including protective pre-treatment, immunoprecipitation, and kinase assay) was validated in primary samples from Ph^+^ leukemia patients, confirming the protection of BCR::ABL1, and from the measurement of kinase activity from immuno-precipitated material (**Figure 2B**). Interestingly, chronic phase (CP)-CML patient samples showed a barely detectable BCR::ABL1 kinase activity compared to the K562 cell line, representing a blast crisis CML. To verify that the reduced activity measured in CP-CML was not a general property of BCR::ABL1 isolated from primary cells, we performed the same analysis on BCR::ABL1 immuno-precipitated from Ph^+^ acute lymphoblastic leukemia samples (Ph^+^ ALL) in a series of samples expressing both p210 and p190 protein variants. Despite similar levels of total BCR::ABL1 protein and Y245 phosphorylation, the kinase activity observed for Ph^+^ ALL was much higher than for CP-CML, comparable to the activity seen in the K562 cell line (**Figure 2C**).

## Discussion

Based on these data, we speculate that a previously unidentified kinase inhibitory mechanism may be active in the early phase of CML, for which the biochemical basis deserves further investigation. This observation could also explain the relatively indolent evolution of the chronic phase of CML that gives rise to peripheral blood leukocytes that preserve nearly normal differentiation and function, which is then lost as a part of the evolution to the later stages of the disease. We are currently exploring this in more detail.

Overall, our data demonstrate that, under native lysis conditions, the substantial degradation of BCR::ABL1 and other critical signaling proteins preclude their recovery from primary samples but that the conditions that we developed for pre-treatment with a protective buffer (the R+L pre-treatment protocol) can rescue that recovery. This suggests the need to apply this protocol for a comprehensive analysis of a functional cell proteome under native lysis conditions. Measuring kinase activity in protease-protected lysates opens the possibility to measure the kinetic parameters of specific kinases derived from protease-rich primary cells from both *ex vivo*- and *in vivo*-treated conditions, which has not been feasible previously. This method has considerable relevance in CML and, in general, for defining an intact and comprehensive protein/phosphoprotein landscape in both healthy and diseased leukocytes.

## Data Availability Statement

The original contributions presented in the study are included in the article/**Supplementary Material**. Further inquiries can be directed to the corresponding author.

## Ethics Statement

The studies involving human participants were reviewed and approved by SPRINT, protocol 1053CESC (Comitato etico per la sperimentazione clinica delle province di Verona e Rovigo). The patients/participants provided their written informed consent to participate in this study.

## Author Contributions

All authors contributed to the article and approved the submitted version.

## Conflict of Interest

The authors declare that the research was conducted in the absence of any commercial or financial relationships that could be construed as a potential conflict of interest.

## Publisher’s Note

All claims expressed in this article are solely those of the authors and do not necessarily represent those of their affiliated organizations, or those of the publisher, the editors and the reviewers. Any product that may be evaluated in this article, or claim that may be made by its manufacturer, is not guaranteed or endorsed by the publisher.
