# Supplementary material for: Successful Preservation of Native BCR::ABL1 in Chronic Myeloid Leukemia Primary Leukocytes Reveals a Reduced Kinase Activity
Source: Front Oncol. 2022 Jun 8;12:904510. doi: 10.3389/fonc.2022.904510 (PMC9216732; doi:10.3389/fonc.2022.904510)
Supplement: Supplementary file 1 [file DataSheet_1.docx]

**Supplementary Information**

**Optical density detection**

Densitometry values of blotted bands were quantified using the ImageJ software and then normalized with indicated housekeeping proteins. OD values for BCR::ABL1 immunoprecipitated from primary samples were standardized in ImageJ software using a calibration curve derived from different amounts of BCR::ABL1 immunoprecipitated from K562 cells, in order to calculate the corrected values (Figure S1).

Figure S1 Calibration curve obtained from immunoprecipitated K562 lysates. Y-axis represent the values (1.56 - 3.12 - 6.25 - 12.5) in µg lysate of K562 from which BCR::ABL1 was immunoprecipitated, while on the X-axis the corresponding densitometric values were indicated.


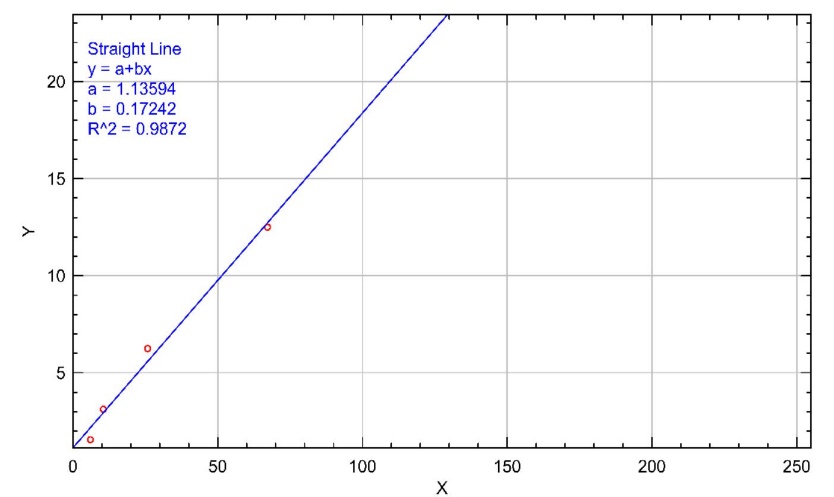


1. Placzek, E.A., et al., *A peptide biosensor for detecting intracellular Abl kinase activity using matrix-assisted laser desorption/ionization time-of-flight mass spectrometry.* Anal Biochem, 2010. **397**(1): p. 73-8.

2. Yang, T.Y., et al., *A multiple reaction monitoring (MRM) method to detect Bcr-Abl kinase activity in CML using a peptide biosensor.* PLoS One, 2013. **8**(2): p. e56627.
